# Audit of antibiotic prophylaxis at a district general hospital

**DOI:** 10.1186/cc14079

**Published:** 2014-12-03

**Authors:** K McGrath, J King-Robson, A Hayes, F Dashti, S Patel

**Affiliations:** 1 Department of Microbiology, Kingston Hospital NHS Trust, Kingston-upon-Thames, UK

## Introduction

This audit evaluates the adherence to the prophylactic surgical antibiotic policy at Kingston Hospital. With a greater understanding of the current use of prophylactic surgical antibiotics comes the ability to improve patient care while minimising the development of antibiotic resistance. Surgical site infections are a major source of hospital-acquired infections, causing significant morbidity and mortality. In appropriate cases, surgical antibiotic prophylaxis is essential in preventing such infections; however, this comes with increased risks of antibiotic resistance and antibiotic-associated diarrhoea. Consequently, this institution has extensive guidelines as to the cases in which antibiotic prophylaxis is required and what antibiotics should be administered. This audit examines the adherence to these guidelines. We audited against our local antibiotic prescribing policy, named the Blue Book guidelines.

## Methods

Notes were audited retrospectively for 80 patients undergoing surgery between 19 August and 18 December 2013. These were audited against local antibiotic guidelines (Blue Book guidelines) and analysed using descriptive statistics.

## Results

Only 57% of operations were compliant with Blue Book antibiotic prophylaxis guidelines. For operations where administration of antibiotics was appropriate, 24% of patients received the incorrect choice, 19% were given the incorrect dose, 28% were given antibiotics for an inappropriate time relative to the procedure, and 38% received antibiotics for the incorrect duration. For operations not requiring prophylaxis, 48% of patients incorrectly received antibiotics.

## Conclusion

Compliance to local guidelines for prophylactic antibiotics is extremely poor. This not only risks increased morbidity and mortality from surgical site infections but also risks the development of antibiotic resistance and *Clostridium difficile *colitis.

We suggest enhancement of the antibiotic guidelines, and education of those using them. This will improve the provision of antibiotic prophylaxis in the Trust, and ultimately improve patient care.

